# Severe vascular complications after derotational osteotomy of the tibia salvaged with free functional latissimus dorsi muscle transfer. A case report

**DOI:** 10.1016/j.jpra.2024.09.010

**Published:** 2024-09-19

**Authors:** T. de Jong, N. van Alfen, R.J. van Heerwaarden, E.T. Walbeehm, T.H.J. Nijhuis

**Affiliations:** a Radboud Peripheral Nerve Centre, Department of Plastic and Reconstructive Surgery, Radboud university medical center, Nijmegen, the Netherlands; b Department of Neurology and Clinical Neurophysiology, Donders Center for Neuroscience, Radboud university medical center, Nijmegen, the Netherlands; c Centre for Deformity Correction and Joint Preserving Surgery, ViaSana Clinic, Mill, the Netherlands; d Department of Plastic, Reconstructive and Hand Surgery, HAGA Hospital, Den Hague and Zoetermeer and Xpert Clinics, the Netherlands

**Keywords:** Foot drop, Neurotization, Free functional muscle transfers, Latissimus dorsi muscle

## Abstract

We present a case study of a 26-year-old male who sustained severe vascular and neurogenic injury during derotational osteotomy of the tibia. Directly postoperatively he complained of a drop foot, but 3 days later presented with an ischemic compartment syndrome of the anterior and lateral compartments. After debridement the osteotomy and metalware were exposed and the patient had a drop foot. Here we report how we salvaged his lower limb with a free functional latissimus dorsi muscle transfer that reconstructed soft tissues and restored ankle dorsiflexion.

## Introduction

Rotational malalignment of the lower leg caused by external tibial torsion is a congenital anatomic variation that does not always reverse spontaneously during growth, and can lead to lower extremity kinetic chain abnormalities in adult life.^1^^,^^2^ Derotation osteotomy of the tibia is an accepted treatment for this condition. A proximal osteotomy has the advantages of more rapid union and a decreased incidence of skin injuries following the procedure.^1^^,^^3^, ^4^, ^5^ However, the proximity of the fibular nerve is a concern. The overall complication rate is 9–13 %, with nerve injury reported in 2.3–2.7 %.^1^

Here we present a case of iatrogenic vascular and neurogenic injury leading to an ischemic compartment syndrome of the anterior and lateral compartment of the lower leg following a proximal derotation osteotomy of the tibia, resulting in a major soft tissue defect, exposed metalware and osteotomy and drop foot. A functional reconstruction was performed with a free neurotized latissimus dorsi muscle transfer.

The patient was consented and agreed to submission of the case for publication.

## Case report

A healthy 26-year-old male underwent a 15° internally rotating proximal tibial osteotomy for a congenital tibial external torsion deformity of the right lower leg. To prevent nerve damage, the nerve was explored at the lateral distal femur and at the fibular head. Immediately following the surgery, the patient noted a foot drop. This was accepted by the operating surgeon because of the preventive measures taken for nerve injury. The patient was discharged the day following surgery. On day 3 the patient was readmitted due to increasing and intolerable pain in the anterior compartment region. Consequently, a two-incision four compartment fasciotomy was performed in the emergency theatre under the assumption of a compartment syndrome. Macroscopically, the muscles in all four compartments were vital, with a capacity to bleed and contractions were seen using cautery. The patient was transferred to the department of plastic surgery in a tertiary referral center for further workup and treatment. To exclude partial (i.e. fascicular) nerve transection, high-resolution ultrasound of the common fibular nerve and its end branches was performed. This showed intact nerve anatomy at the level of the knee joint and fibular head and neck, but a severe, hypoechogenic nerve swelling for several centimeters with uncertain superficial continuity at the level of the fibular shaft, 10 cm below the knee joint (supplement 1). On day 9 a third look was performed by the plastic surgery team with visual confirmation of peroneal nerve continuity and a segmental nerve crush injury with an intraneural hematoma . After debridement of the necrotic parts of the muscles in the anterior compartment the remaining muscle tissue was covered using Split thickness Skin Graft (SSG). On day 28 the patient presented to the outpatient clinic with progressive necrosis of the muscle envelope. Vascular assessment was performed by CTA, that showed an occlusion of the popliteal artery distal to the medial and lateral sural arteries, with close approximation to a screw. The posterior tibial artery was fed by collaterals and no flow was present in the anterior tibial and fibular artery.

**Figure 1:**
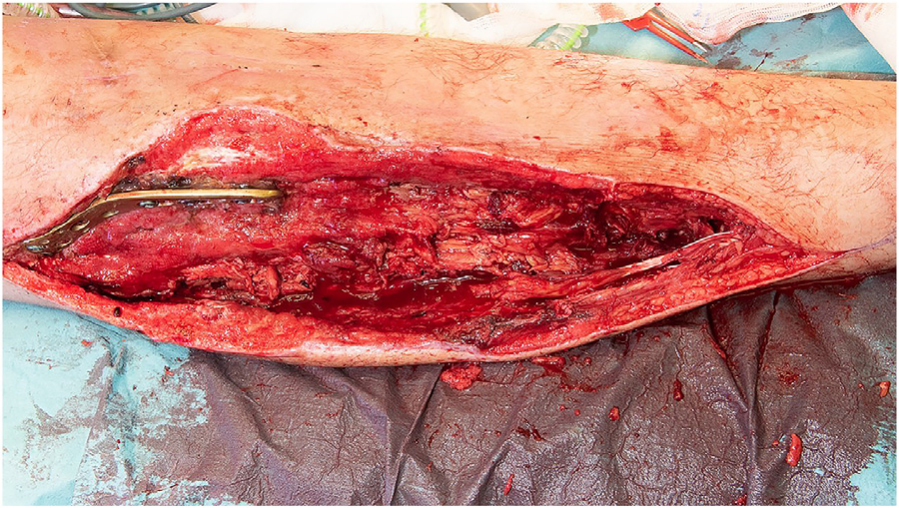
Soft tissue defect after debriding most of the anterior and lateral compartment with metalware and osteotomy exposed.

**Figure 2:**
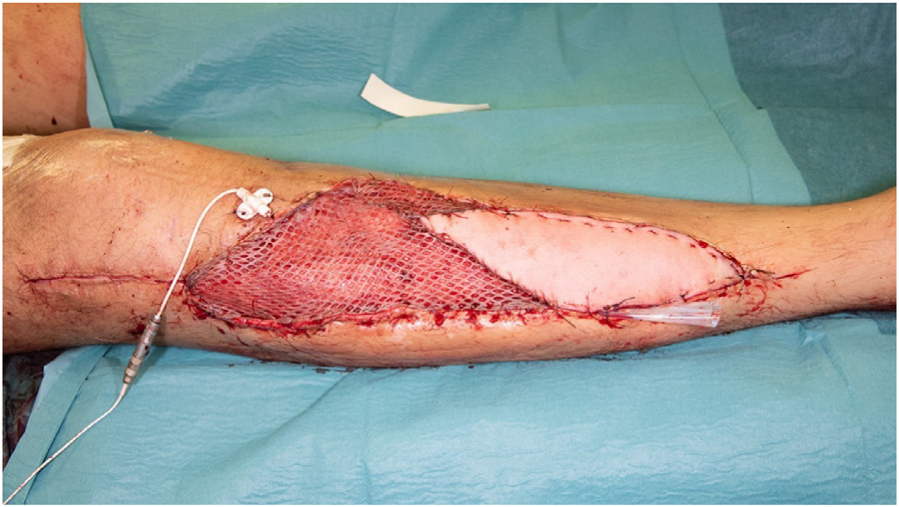
Direct postoperative result after reconstruction with functional latissimus dorsi muscle.

**Figure 3:**
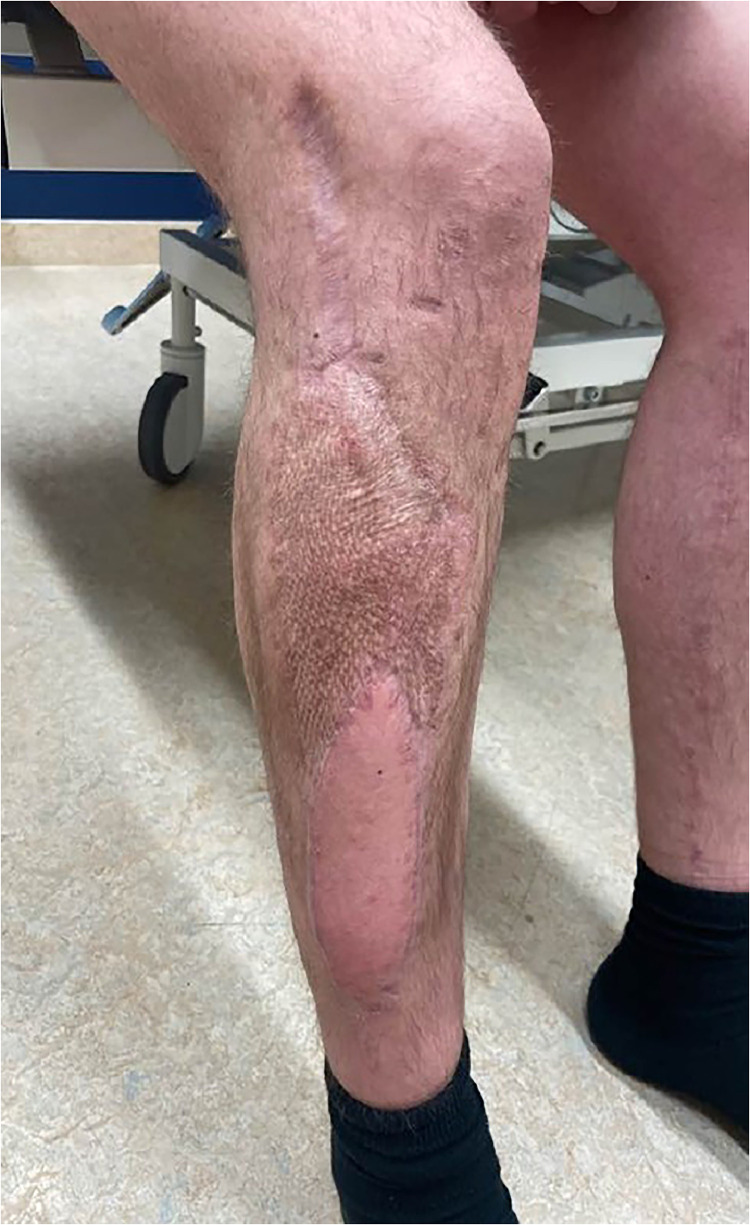
Good soft tissue and aesthetic result 1-year post-surgery.

After an unsuccessful revascularization attempt by the vascular surgery team, the ischemic anterior and lateral compartment muscles were debrided, leaving the patient with tibia osteotomy and osteosynthesis material without proficient soft tissue coverage and the inability of dorsiflexion of the ankle and toes (Figure 1). The following week his lower leg was reconstructed with a free functional latissimus dorsi muscle transfer to provide adequate soft tissue coverage and active foot dorsiflexion. The muscle was rolled up, with a skin paddle inserted in the previous fasciotomy wound for flap monitoring, the proximal end of the muscle was fixed on the tibia plateau and distally the remaining tendons were woven through the muscle, making sure the tension was right (Figure 2). The thoracodorsal nerve was coaptated end-to-end to the unaffected part of the common peroneal nerve at the level of the neck of the fibula for neural reanimation. For the vascular supply the thoracodorsal artery was anastomosed to the femoral artery in Hunter's canal with a vein graft from the great saphenous vein (GSV) of the distal lower leg while the proximal GSV was mobilized and directly anastomosed to the thoracodorsal vein with a coupler device (Synovis GEM coupler). Postoperatively his foot was put in a splint with 10° of dorsiflexion of the ankle for 6 weeks, thereafter an ankle foot orthosis was used until he regained enough strength to go without. One year later his soft tissues have healed without complications and he regained MRC grade 4 dorsi flexion strength (Figure 3 and Video 1).

## Discussion

This case illustrates the impact of iatrogenic neurogenic and vascular injuries during an elective orthopedic procedure resulting in ischemic compartment syndrome of the anterior and lateral compartment of the lower leg, and how a free functional muscle transfer can reconstruct the soft tissues and help regain some of the function lost.

The drop foot directly postoperative was probably due to direct compression or traction damage to the peroneal nerve. Because the correction osteotomy involved internally rotating the tibia 15°, some degree of traction on the nerve was expected.^1^ Therefore the foot drop directly following surgery was “accepted” as a normal immediate post-operative course and monitored closely. The patient presented 3 days after surgery with increasing and debilitating pain. The late presentation of ischemic pain was probably because the compartment syndrome developed slowly due to the still available but minimal blood supply from the medial and lateral sural arteries, that branched of proximal to the occlusion in the popliteal artery.

After debridement this patient needed soft tissue for coverage, to obliterate dead space, provide vascularized tissue to prevent infections and aid bone healing. For major soft tissue defects of the lower extremity several microsurgical reconstructions options are available, including anterolateral thigh, deep inferior epigastric perforator, and latissimus dorsi flap.

Moreover, debridement of the anterior compartment muscles resulted in no chance for recovery of the drop foot. In most cases of foot drop ankle foot orthoses or surgical anterior transfer of tibialis posterior tendon are the preferred choice in management, increasing the mobility and self-independency. In this case the deep posterior compartment might have suffered from a certain degree of ischemia too, making the tibialis posterior muscle an unreliable option for tendon transfer. As the patient also needed soft tissue reconstruction in the form of a free flap, a free functional latissimus dorsi flap was considered as best reconstructive option. The latissimus dorsi muscle has a reliable anatomy and is well described for functional reconstructions of shoulder, biceps, triceps, quadriceps and abdominal wall,^6^, ^7^, ^8^, ^9^ and has once been described for functional reconstruction of the superficial dorsal compartment muscles and overlying skin in the lower leg.^10^ Only 2 cases of free functional muscle transfer for reanimation of dorsiflexion of the ankle seem to have been described in the literature so far, both were pediatric patients that underwent reconstruction with a free neurotized gracillis muscle flap, with a good long-term functional outcome.^11^

In conclusion, iatrogenic vascular injury is a rare but limb threatening condition. Moreover, our case highlights the inherent mechanical risks of soft tissue retraction for the nerves, and illustrates how free functional muscle flaps can be used to reconstructed soft tissues and function simultaneously.

## Ethical approval

Not required. The patient was informed of and agreed to submission of the case data for publication.

## Declaration of competing interest

None.

## Appendix Supplementary materials

Video 1. Patient walking on treadmill without splint, 1-year after reconstruction. Image, video 1

Supplement 1. longitudinal ultrasound image with annotation of the fibular nerve swelling in the peroneal compartment Image, image 2

## References

[1] Fouilleron N., Marchetti E., Autissier G., Gougeon F., Migaud H., Girard J. (2010). Proximal tibial derotation osteotomy for torsional tibial deformities generating patello-femoral disorders. Orthop Traumatol Surg Res.

[2] Lincoln T.L., Suen P.W. (2003). Common rotational variations in children. J Am Acad Orthop Surg.

[3] Krengel W.F., Staheli L.T. (1992). Tibial rotational osteotomy for idiopathic torsion. A comparison of the proximal and distal osteotomy levels. Clin Orthop Relat Res.

[4] van Heerwaarden R.J.K.P., van der Haven I.B, Lobenhoffer RJvH P., Staubli A.E., Jakob R.P. (2008). Osteotomies Around the Knee.

[5] Walton D.M., Liu R.W., Farrow L.D., Thompson G.H. (2012). Proximal tibial derotation osteotomy for torsion of the tibia: A review of 43 cases. J Child Orthop.

[6] Innocenti M., Abed Y.Y., Beltrami G., Delcroix L., Balatri A., Capanna R. (2009). Quadriceps muscle reconstruction with free functioning latissimus dorsi muscle flap after oncological resection. Microsurgery.

[7] Martin S., McBride M., McGarry K., Eames M., Lewis H. (2021). A review of functional latissimus dorsi transfers for absent elbow flexion and supination. Shoulder Elbow.

[8] Muramatsu K., Ihara K., Tominaga Y., Hashimoto T., Taguchi T. (2014). Functional reconstruction of the deltoid muscle following complete resection of musculoskeletal sarcoma. J Plast Reconstr Aesthet Surg.

[9] Ninkovic M., Kronberger P., Harpf C., Rumer A., Anderl H. (1998). Free innervated latissimus dorsi muscle flap for reconstruction of full-thickness abdominal wall defects. Plast Reconstr Surg.

[10] Goertz O., Lehnhardt M., Hirsch T., Stricker I., Steinau H.U., Homann H.H. (2009). Funktionelle Rekonstruktion des M. triceps surae nach onkologiegerechter Tumorresektion beim Kleinkind. Handchir Mikrochir Plast Chir.

[11] Allen L.C.E., Bourke G. (2022). Microsurgical reconstruction for reanimation of foot dorsiflexion in children. Plast Reconstr Surg Glob Open.

