# Supplementary figures and images for: Severe vascular complications after derotational osteotomy of the tibia salvaged with free functional latissimus dorsi muscle transfer. A case report
Source: JPRAS Open. 2024 Sep 19;42:208–12. doi: 10.1016/j.jpra.2024.09.010 (PMC11470784; doi:10.1016/j.jpra.2024.09.010)

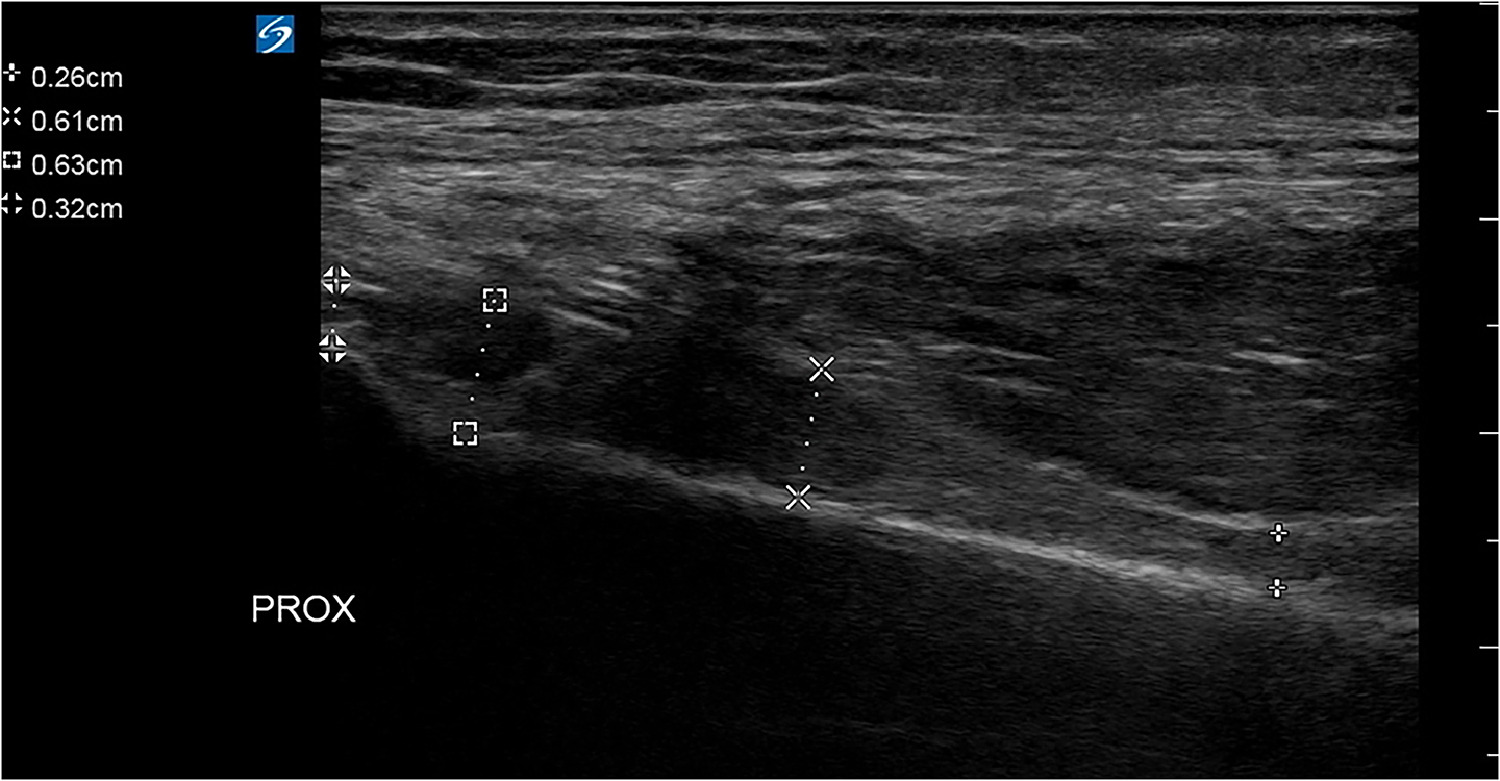

Supplement: Supplementary file 2 — Supplement 1. longitudinal ultrasound image with annotation of the fibular nerve swelling in the peroneal compartment [file mmc2.jpg]
